# Reverse Genetics in Ecological Research

**DOI:** 10.1371/journal.pone.0001543

**Published:** 2008-02-06

**Authors:** Jens Schwachtje, Susan Kutschbach, Ian T. Baldwin

**Affiliations:** Department of Molecular Ecology, Max-Planck-Institute for Chemical Ecology, Jena, Germany; University of Chicago, United States of America

## Abstract

By precisely manipulating the expression of individual genetic elements thought to be important for ecological performance, reverse genetics has the potential to revolutionize plant ecology. However, untested concerns about possible side-effects of the transformation technique, caused by *Agrobacterium* infection and tissue culture, on plant performance have stymied research by requiring onerous sample sizes. We compare 5 independently transformed *Nicotiana attenuata* lines harboring empty vector control (EVC) T-DNA lacking silencing information with isogenic wild types (WT), and measured a battery of ecologically relevant traits, known to be important in plant-herbivore interactions: phytohormones, secondary metabolites, growth and fitness parameters under stringent competitive conditions, and transcriptional regulation with microarrays. As a positive control, we included a line silenced in trypsin proteinase inhibitor gene (TPI) expression, a potent anti-herbivore defense known to exact fitness costs in its expression, in the analysis. The experiment was conducted twice, with 10 and 20 biological replicates per genotype. For all parameters, we detected no difference between any EVC and WT lines, but could readily detect a fitness benefit of silencing TPI production. A statistical power analyses revealed that the minimum sample sizes required for detecting significant fitness differences between EVC and WT was 2–3 orders of magnitude larger than the 10 replicates required to detect a fitness effect of TPI silencing. We conclude that possible side-effects of transformation are far too low to obfuscate the study of ecologically relevant phenotypes.

## Introduction

Reverse genetics, the creation of a phenotype by gene silencing, has rapidly become the method of choice among physiologists for understanding the *in vivo* function of genes. Recent and dramatic advances in our understanding of the cellular function of small RNAs (siRNAs, miRNAs, etc.) in regulating gene expression have allowed for the development of transformation constructs (RNAi, inverted-repeat, antisense, artificial miRNA, etc.), which silence genes with great precision. With the appropriate choice of sequence, constructs can specifically silence individual genes in large gene families as long as a unique 22–24 bp sequence can be identified. At the other extreme, all of the members of a gene family can be silenced with constructs harboring 22–24 bp stretches of sequence shared by all family members [1], [2]. These constructs are easily designed and can be stably or transiently introduced into the genomes of any organism for which transformation systems are available.

In contrast to their rapid adoption by physiologists, these reverse genetic tools have been adopted more slowly by ecologists and evolutionary biologists in their work on the whole-organismic and ecological consequences of gene function. The reasons are likely two-fold: first, a majority of researchers believe that in the evolution of the phenotype, quantitative trail loci (QTLs) are more important than known protein-coding loci and consequently have relied on quantitative genetic techniques to identify QTLs. Second, potential genomic side-effects caused by the transformation process are widely believed to obfuscate the functional analysis of genes that are responsible for specific traits [3]. Dramatic advances in our understanding of the molecular control of complex multi-genic traits have rapidly made the first concern a non-issue. The second, however, remains, and questions regarding how best to control for potential side-effects are relevant.

It is relatively easy to identify genomic effects that result from transformation procedures; many examples have been reported with the widely used *Agrobacterium* transformation system used to infect plant tissue with disarmed *A. tumefaciens* bacteria that integrate modified vector plasmids into the plant's nuclear genome [4], [5]. The transferred DNA (T-DNA) is modified to include the sequence required for silencing the endogenous gene and a selective marker, most commonly an antibiotic resistance gene. *A. tumefaciens* appears to integrate T-DNA into random sites in the nuclear genome, and the insertion process may alter chromosome architecture or DNA sequence. Loss of gene function could result from the insertion of T-DNA into functional gene sequences, or the T-DNA may have pleiotropic effects on the expression of other genes [6], [7]. In addition, genomic changes can result from the tissue culture procedures that are required to transform several plant species, leading to epigenetic and heritable (somaclonal) variation of nucleic DNA [8]–[10]. Clearly, transformation-related alterations of DNA occur to different degrees [7]; yet surprisingly few studies have examined their consequences for whole-plant traits, such as fitness and ecological performance (measured by growth and reproduction, defense and tolerance, and competitive ability). If these reverse genetic procedures are to be used in ecological research, knowing how to most efficiently determine whether these unintended side-effects of transformation confound the analysis of the effects of the targeted gene will be crucial. The question is a quantitative one that requires balancing the number of independently transformed lines against the magnitude of the expected fitness effects. When genes are studied that result in fitness effects sufficiently large to be easily quantified over short time scales, e.g. one growing season, and in experiments with low numbers of replicates, minor molecular changes resulting from the transformation procedure are unlikely to be influential, if the necessary precautions have been followed with transformation procedure.

Several controls and precautions are commonly used to reduce the risk of confounding the unintended effects of transformation with the effects of silencing the expression of a given gene. Insertional mutations can be minimized when plants carry only a single T-DNA insertion. Some vector constructs allow the location of the T-DNA in the nuclear genome to be identified and determine whether a particular gene has been disrupted. An effective but more laborious strategy to rule out unintended effects is to use several independently transformed lines. If T-DNA insertion occurs at random places in the genome, the chances of disrupting the same gene or even different genes which confound the expected RNAi-mediated phenotype in a similar fashion in two independent transformations is extremely small. Backcrossing transgenic plants to their wild-type (WT) parents will also minimize possible transformation effects. However, not all of the variance that results from the transformation procedure is unhelpful for ecological analyses. For example, the variation in transgene expression resulting from inserting the T-DNA into different parts of the genome which have different levels of transcriptional activity (the so-called “positional effects”) can result in lines in which genes are silenced with different degrees of efficiency, despite being transformed with the same T-DNA construct [11]. This variation can be particularly useful for ecological studies: the accumulation of transcripts of the targeted gene can be quantified in several independently transformed lines, not only to demonstrate that the targeted gene was in fact silenced but also to examine the quantitative relationship between gene expression and phenotype. Moreover, including plants transformed with an empty vector construct as controls–plants which have undergone the transformation procedure and carry a T-DNA that lacks the information for silencing of a specific gene but contains all other information necessary for gene silencing, including the antibiotic resistance gene–is thought to be essential for the analysis of transformed plants.

Here we examine the general issue of how many empty vector controls (EVCs) are necessary to estimate the potential unintended effects of the transformation process for ecologically relevant traits. We ask whether the procedure used in our laboratory to transform *Nicotiana attenuata*, a native annual from North America, results in unintended effects on a suite of ecologically relevant herbivore-resistance traits, plant growth and reproductive performance traits. We use plants that have experienced tissue culture and *A. tumefaciens* infection, and that carry a single insertion of an empty vector T-DNA, including a hygromycin-resistance gene. Five independently transformed homozygous EVC lines are compared with isogenic wild types of the same generation of *N. attenuata* in a competition experimental design optimized to identify subtle differences in growth and fitness. The experimental set-up, which involves competing two size-matched seedlings in a single 2 L pot, was designed to simulate the competition for soil nutrients and water that occurs for *N. attenuata* as it germinates synchronously from long-lived seed banks after fires, such as characterize its natural niche in the Great Basin Desert of North America [12]. The competition experiments were carried out two times, with 20 and 10 replicates. The application of methyl jasmonate (MJ), a standardized and reproducible treatment, is known to elicit herbivore defense responses [13]–[16]. We quantified traits that provide demonstrably useful proxies for plant fitness in competitive (height and seed capsule production) and herbivore-intensive environments [phytohormones (JA, JA-Ile) responsible for eliciting the plant's direct defenses, nicotine, and trypsin proteinase inhibitor (TPI) activity], and we hybridized microarrays for a large-scale analysis of potential transcriptional effects. To determine the number of replicates required to detect significant changes in fitness parameters resulting from silencing a defense gene, we included as a positive control transgenic plants that had undergone the same transformation procedure as the EVCs but carried a single insertion of an inverted-repeat construct to silence a TPI gene by RNAi. In previous glasshouse studies, when this gene was silenced in a native *N. attenuata* ecotype that produced TPIs, seed capsule production significantly increased, and when TPI expression was restored by transforming an ecotype that was naturally deficient in TPI expression, seed capsule production decreased [17].

## Results

### Reproductive performance and growth

**Figure 1:**
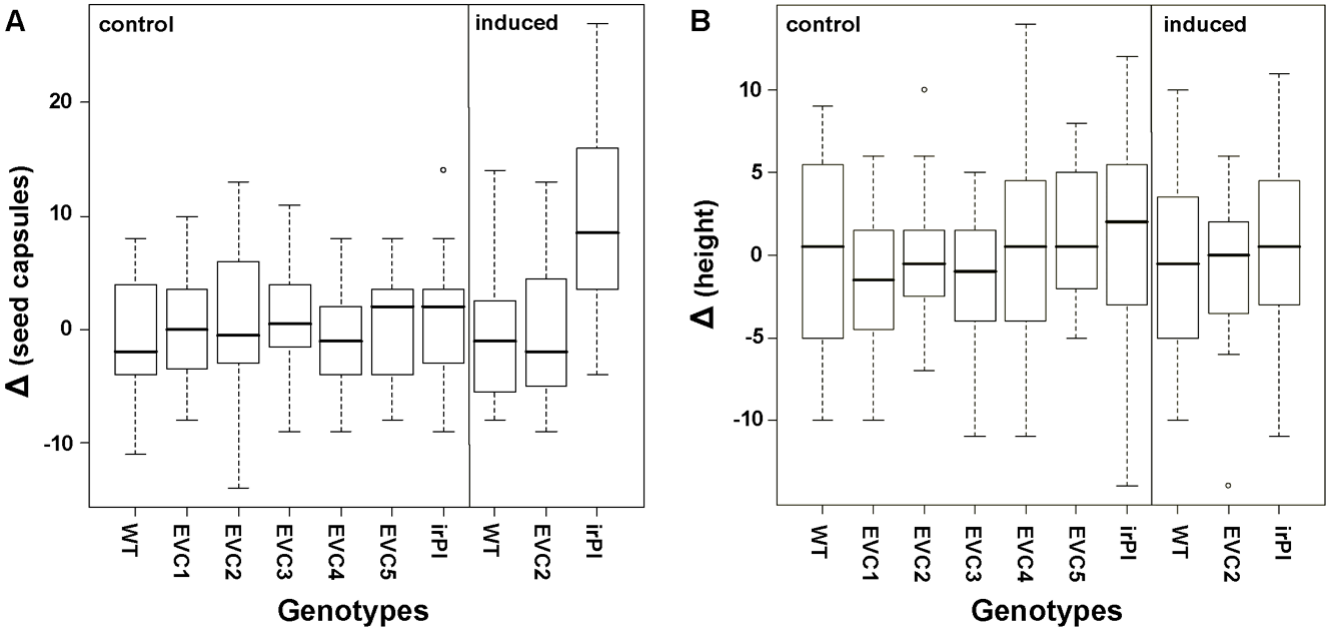
A, B: Differences of seed capsules and stalk heights. Box-plots of differences (Δ) in (A) lifetime seed capsules (number) and (B) height (cm), among pairs of initially size-matched plants competing in a 2-L pot (control: untreated; induced: MJ-treated). Every isogenic plant of a genotype noted on the x-axis was paired with an isogenic WT plant. N = 20; for statistical evaluation see text. Whiskers designate the 95 % confidence interval of data.

Results from previous studies with *N. attenuata* plants silenced and ectopically expressing TPIs [17] revealed that in competition experiments, 10 replicates are sufficient to reveal significant differences in lifetime seed capsule production among TPI-expressing and TPI-silenced pairs. No significant differences were found between WT and EVC pairs in the previous analyses with paired t-tests. Here we increased our statistical power to detect fitness-related differences among WT and EVC pairs by using 20 replicates. Each of the 5 EVC lines, the irPI line and WT were paired with WT in a 2 L pot after being size matched. The paired design was chosen to impose competition on plant pairs: the limited resources of a 2L pot strongly amplify small differences in growth rates, resource allocation, or competitive ability between the initially same-sized plants [18]. For the three genotype pairings that were elicited with MJ (see Fig. 1), we elicited the plants twice to increase defense responses and their associated costs, to increase the chance of detecting spurious plant growth and performance effects in the competition set-up. To compare differences between plant pairings (two sample t-test), for each plant pair the two values of a measured trait were subtracted from each other, and the mean difference for all replicates of a pairing is referred to as Δ, combined with subscripts that describe the measured trait (e. g. Δ_nicotine_). For paired t-tests we compared means of the measured trait of each of the two lines of a pairing.

As expected, we found Δ_seed capsules_ of the irPI-WT pairing to be significantly larger compared to Δ_seed capsules_ of the WT-WT after MJ induction (ANOVA: F_9,190_ = 5.825, *P*<0.0001; post-hoc test: Tukey/Kramer: *P*<0.0001, Fig. 1A), as well as compared to Δ_seed capsules_ of the EVC-WT pairings (post-hoc test: Tukey/Kramer: *P*<0.0001, Fig. 1A); this is because irPI plants produced more seed capsules than did WT and EVC plants. Clearly, silencing this potent defense significantly increases *N. attenuata*'s reproductive performance, a result consistent with a significant cost of TPI production.

No other pairings showed significant differences of Δ_seed capsules_ compared to WT-WT pairings (ANOVA: F_8,171_ = 0.330, *P* = 0.9537). Moreover, no differences in Δ_height_ could be detected (ANOVA: F_9,190_ =  0.863, *P* = 0.5594, Fig. 1B). These results demonstrate that none of the 5 EVCs differed from WT plants in any growth-related measure in a competition design which is designed to detect smaller differences in performance than those that can be detected when plants are grown in single pots [19].

Paired t-tests for seed capsules of MJ treated plants resulted in *t* = −0.223; *P* = 0.8257; DF = 19 for EVC-WT pairings, in *t* = 5.276; *P*<0.0001; DF = 19 for irPI-WT pairings, and for the WT-WT pairings in *t* = −0.419; *P* = 0.68; DF = 19. In a second competition experiment, replicated 10 times and with the same pairings as the first experiment, plants were grown during the short-day season of the year; this schedule resulted in lower absolute amounts of seed capsules per plant (20 % fewer; 39.3 vs. 47.2 seed capsules on average) than in the experiment with 20 replicates. Paired t-tests for this experiment for seed capsules of MJ treated plants resulted in *t* = −0.072; *P* = 0.9444; DF = 9 for EVC-WT pairings, in *t* = 2.613; *P* = 0.0281; DF = 9 for irPI-WT pairings, and in *t* = 0.196; *P* = 0.8489; DF = 9 for WT-WT pairings.

### Power analyses

To determine the minimum sample sizes necessary to detect significant differences between Δ_seed capsules _of irPI-WT and WT-WT, and between Δ_seed capsules _of EVC-WT and WT-WT (two sample t-test), power analyses (for β≤0.2) were carried out with the data from the two experiments (MJ-treated plants). In addition, power analyses (for β≤0.2) were carried out based on paired t-tests (mean difference of one pairing), as this is the statistical test we use to analyze performance effects of transformed plants in glasshouse competition experiments as well as in field trials [17], [20].

**Figure 2:**
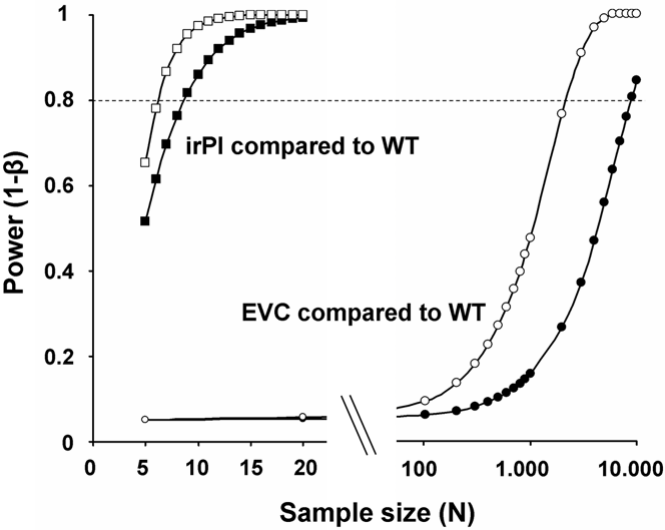
Power analysis for Δ_seed capsules_. Calculated statistical power (dotted line: power = 0.8) as a function of sample size for detection of significant differences for Δ_seed capsules_ (two sample t-test) between irPI-WT and WT-WT (closed squares), and EVC-WT and WT-WT (closed circles), and for significant differences between two means (paired t-test) of irPI-WT (open squares) and EVC-WT (open circles), based on seed capsule data from the first experiment (N = 20). Note that the scale of the x-axis is diverted in a linear (left) and a logarithmic (right) segment.

Calculated sample sizes necessary to detect differences between Δ_seed capsules _of EVC-WT and WT-WT, based on the experiment with 20 replicates, resulted in N = 8865 (1−β = 0.8, Fig. 2), and calculated sample sizes necessary to detect significant differences between Δ_seed capsules_ of irPI-WT and WT-WT resulted in N = 9 (1−β = 0.82, Fig. 2). Based on the two means of a paired t-test, least sample size for the irPI-WT pairing resulted in N = 7 (1−β = 0.866), for the EVC-WT pairing in N = 2186 (1−β = 0.8), and in N = 2536 (1−β = 0.8) for the WT-WT pairing.

For the second competition experiment with 10 replicates, sample size calculation for differences between Δ_seed capsules_ of EVC-WT and WT-WT resulted in N = 2602 (1−β =  0.8) and for differences between Δ_seed capsules_ of irPI-WT and WT-WT in N = 17 (1−β = 0.81). Based on a paired t-test least sample sizes for the EVC-WT pairing were N = 8718 (1−β = 0.8), and for the irPI-WT pairing N = 13 (1−β = 0.828).

### Nicotine levels and TPI activity

**Figure 3:**
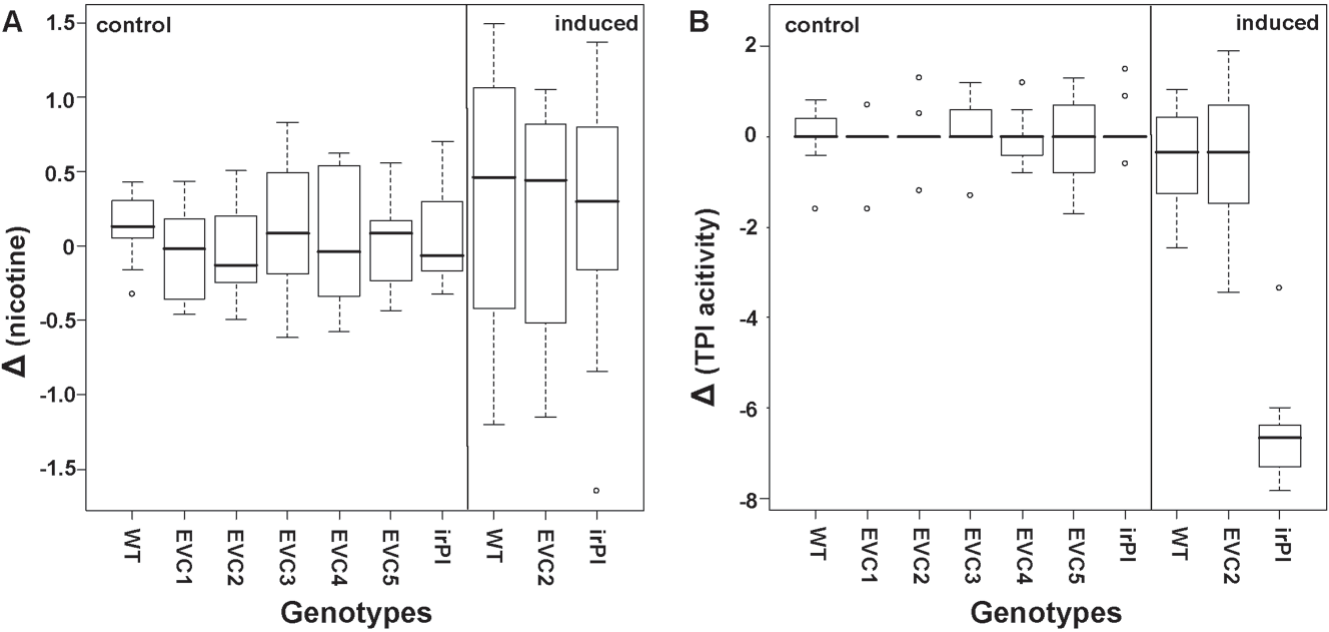
A, B: Differences of nicotine and TPI activity. Box-plots of differences (Δ) in (A) nicotine content (mg/g fresh mass) and (B) PI activity (nmol/mg protein) among pairs of initially size-matched plants competing in a 2-L pot (control: untreated; induced: MJ-treated). Every isogenic plant of a genotype noted on the x-axis was paired with an isogenic WT plant. N = 10; for statistical evaluation see text. Whiskers designate the 95 % confidence interval of data.

Nicotine levels and TPI activity were measured in the 10 replicate experiment. A Levene-test revealed no homoscedasticity of Δ_nicotine_ and Δ_TPI activity_ for control and induced pairings; thus, these two groups were analyzed separately. Levene-tests for control (uninduced) pairings were non-significant for Δ_nicotine_ (F_6,63_ = 1.404, *P* = 0.238) and Δ_TPI activity_ (F_6,63_ = 0.729, *P* = 0.607). We did not find significant differences between the Δ_nicotine_ of uninduced plants in any pairing (ANOVA: F_6,63_ = 0.352; *P* = 0.954, Fig. 3A), and Δ_TPI activity_ (ANOVA: F_6,63_ = 0.179, *P* = 0.982, Fig. 3B). MJ induction did not influence the homoscedasticity of Δ_nicotine_ and the Δ_TPI activity_ of plant pairs (Levene-test: nicotine: F_2,27_ = 0.180, *P* = 0.838; TPI activity: F_2,27_ = 1.169, *P* = 0.326). Δ_nicotine_ was not different between induced pairings (ANOVA: F_2,27_ = 0.046, *P* = 0.956, Fig. 3A), but Δ_TPI activity_ was significantly different (ANOVA: F_2,27_ = 70.19, *P*<0.0001; post-hoc test: Tukey/Kramer for irPI-WT and WT-WT pairings: *P*<0.0001, for EVC2-WT and irPI-WT pairings: *P*<0.0001, for EVC2-WT and WT-WT pairings: *P* = 0.997, Fig. 3B). As expected, the TPI activity of MJ-induced irPI plants was not detectable [21], demonstrating that the TPI gene had been effectively silenced by the transformation with the inverted-repeat construct.

### Microarrays

**Figure 4:**
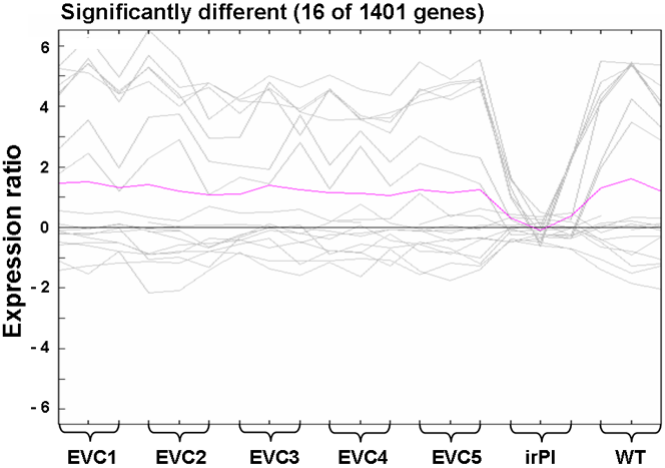
Gene expression ratios. Graph of expression ratios (log_2_ transformed) of significantly different expressed genes in source leaves of each genotype 24 h after elicitation with MJ. Microarrays were hybridized with three different biological replicates for each genotype (the MJ-treated sample was labeled with cy3, controls with cy5). ANOVA, 2000 permutations, α: 0.01. For a list of different and non-different genes, see Supplemental Information.

We hybridized 21 microarrays, consisting of three biological replicates, for all 7 genotypes, to determine if transcript accumulation 24 h after treatment with MJ differed among the genotypes. An ANOVA (2000 permutations, alpha: 0.01) revealed 16 significantly differently regulated genes (including 7 PI genes), most of them in the irPI microarrays (Fig. 4A, for a list of genes, see Table S1). Most genes (1385) were not differentially regulated between plant lines, i.e. they responded similarly to MJ treatment in every plant line (Table S2).

### Hormones

JA and its amino acid conjugates (JA-Ile) play crucial roles in the defense signaling of plants [22], regulating nicotine and TPI production, as well as a variety of other defense traits, such as volatile emission [19]. When plants were elicited by wounding and immediately applying caterpillar regurgitant to the wounds, the elicitation of JA and JA-Ile/Leu conjugates did not differ among all genotypes (JA: ANOVA; F_6,14_: 0.664; *P* = 0.681; JA-Ile/Leu: ANOVA; F_6,14_: 0.997; *P* = 0.465, Fig. S1).

## Discussion

The objective of this study was to evaluate the effect of transformation, based on tissue culturing and *A. tumefaciens* infection, for possible side-effects on ecologically relevant performance traits, in order to determine how many and which controls are needed in functional ecological experiments with gene-silenced *N. attenuata* plants. This issue is of particular importance for ecologists, because if the power of reverse genetics is to be realized for field studies, where large sample sizes can be onerous, it is important to determine the effort that is required to evaluate the possibility that side-effects of the transformation process confound the performance analysis of a gene of interest.

We examined the performance of 5 independently transformed EVC lines in comparison to their isogenic WT counterparts of *N. attenuata*, with a variety of ecologically relevant traits including growth, reproductive output, defense metabolites and MJ-elicited transcriptional responses of numerous secondary and primary genes with microarrays. In order to increase our ability to detect subtle differences, a competition set-up was used which paired transformed plants with isogenic WT plants. As a positive control for gene silencing effects, we included pairings with an irPI line that lacks a TPI activity and is known to have significantly increased reproductive performance [17]. In no experiments were significant differences between EVC and WT plants detectable for any measured traits: hormone levels, nicotine levels (Fig. 3A), TPI activity (Fig. 3B), transcriptional profiles of a large number of genes (Fig. 4), height and reproductive (Darwinian) fitness (Fig. 1 A, B). Darwinian fitness, the crucial parameter in functional ecological studies, was estimated by the number of seed capsules produced. This number expresses a plant's fitness through the female function, which gives an accurate fitness estimate especially for this largely self-pollinating species [23], and reflects the total of all effects, positive and negative, on a plant's reproductive capacity during growth under intense competition with an isogenic WT plant. Subtle differences in plant growth are frequently amplified when plants are grown in competition; note for example, the fitness costs associated with MJ elicitation [18]. We conclude that regardless of the unmeasured molecular side-effects that might have occurred during tissue culturing and *Agrobacterium* transformation of EVC lines, none significantly influenced any of these ecologically important traits.

A power analysis (for β≤0.2) of the number of replicates required to detect fitness differences between EVC or irPI and WT lines based on the 20-replicate experiment revealed just how small the chances were of having transformation-related side-effects confound the analysis of TPI expression on reproductive performance. In order to detect differences between EVCs and WT plants, a sample size 3 orders of magnitude larger than that required to detect the effects of TPI-silencing would be necessary (Fig. 2). Based on a paired t-test, differences between competing WT plants could be detected with N = 2536 (1−β = 0.8) and between competing WT and EVC plants with N = 2186 (1−β = 0.8), which clearly demonstrates how similar EVC and WT plants are.

The difference of required sample sizes was less pronounced when the power analysis was conducted with the data from the 10-replicate experiment conducted during the short-day period of the year, where plants produced 20% fewer capsules; however, the TPI effect is still dramatically larger than the differences between EVC and WT.

How many independent EVC lines should be included to test for transformation effects, and how many replicates are necessary to detect significant ecological effects of single genes? To answer this question, the work of Tian *et al.* [24] is often cited as the standard. This study used an elegant Cre/Lox transformation system for *Arabidopsis* with backcrossed controls, a variety of controls that examine the effects of T-DNA insertions, and 500 replicates to measure the large fitness effect of an resistance (R) gene (9 % more seeds in plants lacking the R gene). While this is an exemplary study of the use of transgenic plants to study an ecological question, in our opinion, the study is over-controlled, and inappropriate to use as the standard to which all other studies should be held. The fitness cost of the R-gene could have been detected with many fewer replicates had the research been done as a traditional reverse-genetics study targeting the fitness consequences of a known gene. The sample size requirements of a QTL analysis to detect very small effect sizes are often confused with those required for a reverse-genetics study. Studies which aim to determine QTLs that influence adaptive ecological traits in the long-term context of evolution generally require a large number of replicates for statistically significant effects. Reverse genetics in ecology, however, aims to understand the effect on Darwinian fitness of an established trait, mostly related to one or few genes, and therefore will most often lead to much larger effects, which are comparatively easy to detect with lower numbers of replicates, as our power analysis demonstrated.

How many EVC lines should then be used for experiments in functional ecology? The fact that the differences of EVC and WT plants we describe here are extremely low compared to the great effects of silencing of the TPI gene (Fig. 2) suggests that the use of WT plants as controls is sufficient for ecological experiments with *N. attenuata* and that EVCs can be omitted. Time-consuming procedures, such as repeated backcrossing with WT parents to obtain “clean” transgenes, are not justified by the results of this study. The evaluation of several independently transformed lines is part of tests for side-effects of the transformation procedure. If the same quantifiable phenotype appears at least twice in different lines transformed with the same construct, the probability is extremely low that the phenotype will be a result of unintended side-effects of transformation, especially, when the phenotype can be predicted from the biochemical function of the silenced gene.

Side-effects are often found in purely molecular analysis of transformed organisms, however, only few studies describe their translation to the whole-plant level. Generally, most effects fall in the natural range of variation [25]. One of the most carefully designed studies was done by Purrington and Bergelson [26], who used several independently transformed and double backcrossed EVCs carrying an antibiotic resistance gene. They could show that the seed production of transformed *Arabidopsis* plants engineered for antibiotic resistance did not differ from that of WT controls, and that the expression of the resistance gene is not associated with metabolic costs. This is an important finding, since antibiotic resistance genes are commonly used in reverse genetics. Ruebelt *et al*.[27] found that the differences of 2D seed protein profiles of *Arabidopsis* between wild types and several transformed plants were small and fell in the range of the differences among 12 Arabidopsis ecotypes. Rogan *et al*. [28] examined two types of transgenic virus-resistant potatoes and found them substantially equivalent to wild types within a variety of metabolites, nutrients and general morphological parameters. Another recent study compared various potato lines from two varieties and included WT plants, untransformed plants that have undergone tissue culture, EVCs, and plants with genes in both sense and antisense orientation [29]. Measurements were made of numerous primary and secondary metabolites, as well as of general parameters, such as dry mass and tuber numbers. Some significant but randomly distributed differences were reported among transgenic plants, tissue-cultured plants, and wild types, but none of these could be attributable to a specific construct. The most obvious differences existed between the two potato varieties. However, unintended changes caused by tissue culture appear to be a problem for some species [25]. Sandoval *et al*. [30] observed that dwarf types of Cavendish banana had lower endogenous gibberellin levels, affecting banana plant height, and Ramulu *et al*. [31] described DNA variation in micropropagated potato. However, in our study we did not detect any differences which could be attributed to tissue culturing.

Glasshouse experiments aim to mimic natural growth conditions; however, despite supplemental illumination from HID Na-vapor lamps, the variation of sunlight intensity and day length during the year may influence plant growth. To detect seasonal differences, we compared fitness data from two experiments on plants grown during short- and long-day seasons. When days were shorter, the total number of seed capsules produced per plants was 20 % fewer than from plants grown during a long-day period. With a power analysis, we could demonstrate that gene silencing effects (irPI vs.WT) of both experiments largely outweighed differences between EVC and WT, but that the smallest number of replicates required for significant differences were slightly higher for irPI-WT differences compared to the experiment carried out during the long day season. None of the other measurements, e.g. of nicotine levels and of TPI activity, differed from measurements made in field studies. This demonstrates that the glasshouse environment sufficiently mimics conditions found in the natural environment of *N. attenuata*.

Our study demonstrates that laborious testing of transformed plants does not reveal any significant change in various ecologically relevant plant traits, or in the transcription of numerous ecologically and physiologically relevant genes. Since we generally examine genes that have marked effects on defense and fitness traits, which are detectable with relatively low numbers of replicates, we are convinced that reverse genetics is a powerful and accurate tool for ecological studies and that its potential to obfuscate intended gene silencing are very low. The genome of *Nicotiana* species is large and has a relatively low gene density; for example, the *Nicotiana tabacum* genome is 20 times larger than that of *Arabidopsis thaliana* [32]. The large size of the *N. attenuata* genome may have contributed to our failure to detect side-effects of transformation; gene disruption might be a rare event in a large background of non-coding DNA. The small and compact genome of *Arabidopsis* may lead to a higher probability for transformation-related side-effects and therefore require more stringent controls.

To use transformed plants and reverse genetics in ecological experiments to measure the consequences of gene silencing on plant performance, we suggest the adoption of the following procedures:

A reasonable guess about phenotypic effects of gene silencing can be made when a particular gene with an annotation is silenced. Thus, plants with extreme phenotypes can be easily excluded. Several independently transformed lines of gene-silenced plants should be used and these lines should be homozygous for the T-DNA (homozygosity can only be tested with diploid plants) and carry a single copy of T-DNA. (Southern blot analysis usually suffices for such measurements, although it is not foolproof because fragments of the T-DNA may have been inserted elsewhere in the genome and these may not match the Southern probe). The use of particular transformation vectors with rescue functionality (e.g. [33], [34] make it possible to recover the T-DNA along with right and left border flanking sequence which helps to identify the insertion site and determine whether a known DNA sequence has been disrupted). The possible pleiotropic effects of T-DNA insertions can not be tested with single transformed lines because the random insertion of T-DNA into the genome (e.g. with *Agrobacterium*) makes it likely that each transformed line is different.

The mRNA level of the targeted gene has to be lower in transgenic lines compared to wild type lines. At least two and preferably three transformed lines should meet the above criteria and show similar reductions of mRNA. Lines that differ in gene silencing can be used to correlate phenotypes with mRNA levels.

Initial experiments to measure the effect size of the silenced gene on the phenotype (growth, reproductive performance, the gene-related trait, etc.) should be carried out under conditions that mirror conditions in nature. The effect size allows sample sizes to be calculated by means of a power analysis for further studies, e.g. in the field.

Several independent EVC lines should be generated to test for unintended effects related to the transformation procedure and the specific T-DNA. Growth, morphology, reproductive fitness, and ecological traits that are characteristic of the species should be compared in transformed lines and wild types under environmentally realistic conditions. Reproductive performance reflects all possible side effects of transformation that may disturb a plant's physiology. The use of EVC lines as transformation controls is not necessary when no differences compared to wild types have been measured with experiments with a sample sizes informed by the effect size of the silenced gene. In this situation, the use of wild types as controls is sufficient.

Transformation side effects depend on the gene density of an organism's genome; the lower the gene density, the less probability there is that gene functions will be disturbed by T-DNA insertions.

## Materials and Methods

### Plant transformation and regeneration

We used 5 independently transformed EVC lines containing the pRESC2NC vector construct that lacks the silencing information and carries a hygromycin-resistance gene, as described in [17], [33]. The plants were regenerated from tissue culture after being transformed by *A. tumefaciens* (strain LBA4404, Life Technologies-Gibco BRL) as described in [35]. T_2_ generations were used for the experiments. Transgenic plants were tested for homozygosity (by segregation analysis), diploidy (by flow cytometry), and single insertion of T-DNA (by Southern blotting). Hypocotyls from 8-day-old seedlings germinated on Gamborg's B5 medium were cut with a scalpel into several 3-mm-long pieces after the scalpel had been dipped in a suspension containing the vector-harboring *A. tumefaciens*. On different phytagel-based media, the explants and resulting calli/plants went through five stages: co-cultivation (3 days), callus growth (14–21 days), shoot regeneration (14–21 days), shoot maturation (14–21 days), and rooting (21 days). After rooting, the plants were transferred to soil in Magenta boxes (www.labdepotinc.com) and finally planted in 2L pots in the greenhouse for breeding.

To analyze the phenotypic effects of gene silencing, we used as a positive control an irPI line that had undergone the same transformation procedure and contained an inverted-repeat (ir) silencing sequence for an *N. attenuata* TPI gene as described in [21]. The fitness effects of TPI silencing were similar among plants silenced with antisense and inverted-repeat constructs [36]. All transformed plants stem from a wild-type inbred line of the 14^th^ generation, which was originally collected from a plant growing near Santa Clara, Utah. Transformed plants used for experiments were the T_2_ generation. As isogenic controls, we used wild-type (WT) plants from the 17^th^ generation of the inbred line.

### Plant growth and fitness measures

*N. attenuata* plants were grown in the glasshouse under conditions described in [35], [37] with a 16 h light: 8 h dark period and artificial light (335 µmol*m^−2^ *s^−1^ at 1.5 m distance). For both experiments, plants were potted in 2 L pots containing soil, with two plants per pot. The plant pairings included each of the 5 EVC lines [A-04-08 (EVC1), A-04-09 (EVC2), A-04-104 (EVC3), A-04-110 (EVC4), A-04-117 (EVC5)] paired with WT, irPI (A-04-186) paired with WT, and WT paired with WT. Rosette diameters were measured after plants grew for 8 days in the glasshouse, and for each plant combination, the best-matched pairs were selected.

For fitness measurements in the first experiment, the 20 best-matched pairs (of an initial 28) were chosen from each combination of genotypes. Plants were allowed to elongate fully for a 35-day growing period. At the end of the growing period, watering was reduced over 10 days as described in [38], to simulate the water conditions of *N. attenuata* at the end of the growing season in its natural habitat. After an additional 12 days, when all capsules were fully ripened, height and fitness parameters were measured; the latter was estimated by lifetime seed capsule production. This experiment was carried out during April-June, a season where sunlight and day-length enhance the PAR supplied by supplemental Na-vapor HID lamps of the glasshouse (19,20). In a second competition experiment, conducted during October-December when the PAR provided by natural sunlight is lower, but under the same supplemental lighting as above, we measured nicotine, TPI activity (by sampling one elicited leaf from all plants) and fitness. For this experiment, the 10 best-matched pairs (of 16) were chosen.

### Nicotine levels and TPI activity measurements

At the rosette stage, directly after size matching, 10 pairs of WT-WT, irPI-WT, and EVC2-WT were treated with 150 µg MJ dissolved in 20 µl lanolin as described in [39]. MJ, which is known to elicit defense responses very similar to those elicited by insect herbivory, was applied to the bases of 2 source leaves growing at nodes +1 and +3 [13]–[16]. Three days after MJ treatment, samples were taken from a systemic (non-treated) source leaf of each plant of the 3 induced and 7 control pairings (all 5 EVC, irPI and WT, each paired with WT) and measured for nicotine levels (with HPLC) and TPI activity (with radial diffusion assays) according to procedures described in [40] and [18].

### Hormone measurements

For phytohormone measurements, 6 plants of each genotype were grown individually in 1 L pots and analyzed at the rosette stage of growth. To elicit a change in phytohormone response in a highly reproducible manner and thereby allow subtle changes in phytohormone kinetics to be detected (see [22], for example), one source leaf in half of the plants of each line was wounded with a pattern wheel and 20 µl 1:5 water-diluted oral secretions and regurgitate (OS) from the specialist lepidopteran herbivore *Manduca sexta*, a species that regularly accounts for major losses of leaf area in native populations of *N. attenuata*, was applied. This treatment elicits defense responses similar to those of feeding *M. sexta* [13]. Induced and control samples were harvested 45 min after treatment, when jasmonic acid (JA) levels reach their maximum, and measured by LC/MS/MS according to [41].

### Microarray analysis

For microarray samples as for phytohormone analysis, additional plant pairs were established so that the sampling did not confound the measures of plant performance. Two source leaves per plant were treated at 11 a.m. with 150 µg MJ as described above. Leaves were harvested after 24 h and RNA was extracted as described in [38]. For each of the seven plant lines, 3 microarrays were hybridized to compare transcriptional profiles of elicited and unelicited leaves of plants of the same line. RNA was processed for microarray analysis and microarrays were hybridized as described in [20], [42], [43]. RNA from MJ elicited leaves was labeled with cy3; RNA from the control leaves of unelicited plants of the same line was labeled with cy5. Approximately 400 µg total RNA was used in each labeling reaction. The microarray was enriched with *N. attenuata* genes, which are known to be responsive to *M. sexta* attack [14], [37], [42]–[45]. Microarray data were lowess-normalized with the MIDAS package [46]. The quadruplicate spots of each gene were analyzed for significant differences using a t-test at a confidence level (α) of 0.05, and a threshold of a 1.5-fold change in expression ratio was used. For statistical analysis, all 21 microarrays were analyzed with the TMEV software [46].

### Statistical analysis

Samples were evaluated with a two sample t-test (and a paired t-test). Differences among traits of the paired set-up were analyzed by a Levene-test for homogeneity and subsequent analysis of variances (Model 1 ANOVA). Unless otherwise noted, Levene tests revealed no significant heteroscedasticity. The Tukey/Kramer test was used as a post-hoc test. Statistics were carried out by using Statview (www.jmp.com) and the free statistics software, R (www.r-project.org). For power analyses, we used the statistical software PASS (www.ncss.com).

## Supporting Information

Figure S1 JA levels after elicitation. Levels of JA (upper graph) and JA-Ile/Leu conjugates (lower graph) in source leaves 45 min after elicitation with oral secretions of M. sexta. Means+SE. No statistically significant differences could be detected.
(0.15 MB TIF)

Table S1 Differently regulated genes. Table of microarray data
(0.02 MB XLS)

Table S2 Non regulated genes. Table of microarray data
(0.15 MB XLS)
