# Supplementary figures and images for: Reverse Genetics in Ecological Research
Source: PLoS One. 2008 Feb 6;3(2):e1543. doi: 10.1371/journal.pone.0001543 (PMC2212111; doi:10.1371/journal.pone.0001543)

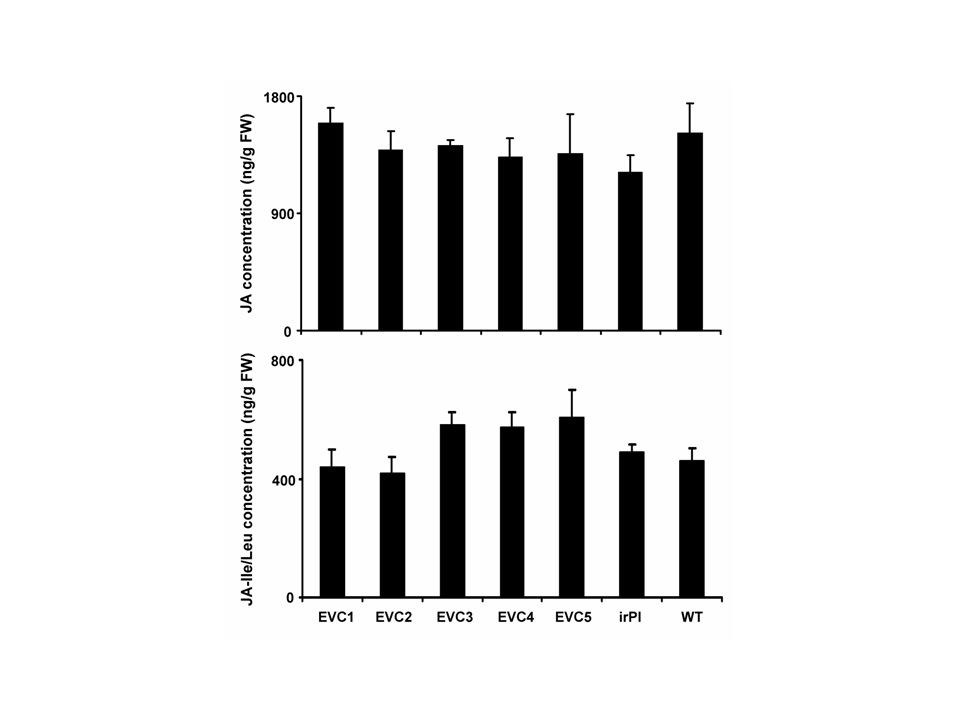

Supplement: Figure S1 — JA levels after elicitation. Levels of JA (upper graph) and JA-Ile/Leu conjugates (lower graph) in source leaves 45 min after elicitation with oral secretions of M. sexta. Means+SE. No statistically significant differences could be detected. (0.15 MB TIF) [file pone.0001543.s001.tif]
